# Genome-wide and comparative phylogenetic analysis of senescence-associated NAC transcription factors in sunflower (*Helianthus annuus*)

**DOI:** 10.1186/s12864-021-08199-5

**Published:** 2021-12-14

**Authors:** Sofia A. Bengoa Luoni, Alberto Cenci, Sebastian Moschen, Salvador Nicosia, Laura M. Radonic, Julia V. Sabio y García, Nicolas B. Langlade, Denis Vile, Cecilia Vazquez Rovere, Paula Fernandez

**Affiliations:** 1 Instituto de Agrobiotecnología y Biología Molecular, INTA-Castelar, Buenos Aires, Argentina; 2 Bioversity International, Montpellier, France; 3 Estación Experimental Agropecuaria Famaillá, INTA-Famaillá, Tucumán, Argentina; 4 Université de Toulouse, INRAE, CNRS, Castanet-Tolosan, France; 5 LEPSE, Université de Montpellier, INRAE, Montpellier, France

**Keywords:** NAC transcriptions factors, Leaf senescence, Sunflower, *Arabidopsis thaliana*, High throughput phenotyping, HaNAC001

## Abstract

**Background:**

Leaf senescence delay impacts positively in grain yield by maintaining the photosynthetic area during the reproductive stage and during grain filling. Therefore a comprehensive understanding of the gene families associated with leaf senescence is essential. NAC transcription factors (TF) form a large plant-specific gene family involved in regulating development, senescence, and responses to biotic and abiotic stresses. The main goal of this work was to identify sunflower NAC TF (HaNAC) and their association with senescence, studying their orthologous to understand possible functional relationships between genes of different species.

**Results:**

To clarify the orthologous relationships, we used an in-depth comparative study of four divergent taxa, in dicots and monocots, with completely sequenced genomes (*Arabidopsis thaliana, Vitis vinifera, Musa acuminata* and *Oryza sativa*). These orthologous groups provide a curated resource for large scale protein sequence annotation of NAC TF. From the 151 HaNAC genes detected in the latest version of the sunflower genome, 50 genes were associated with senescence traits. These genes showed significant differential expression in two contrasting lines according to an RNAseq assay. An assessment of overexpressing the Arabidopsis line for HaNAC001 (a gene of the same orthologous group of *Arabidopsis thaliana* ORE1) revealed that this line displayed a significantly higher number of senescent leaves and a pronounced change in development rate.

**Conclusions:**

This finding suggests HaNAC001 as an interesting candidate to explore the molecular regulation of senescence in sunflower.

**Supplementary Information:**

The online version contains supplementary material available at 10.1186/s12864-021-08199-5.

## Background

Leaf senescence is a complex process controlled by multiple genetic and environmental variables and with strong impact on crop yield [1]. In sunflower, the fourth most important oil crop worldwide, senescence reduces the capacity of plants to maintain their green leaf area for longer periods, thus leading to economic losses [2, 3]. In different crops, including sunflower, a delay in leaf senescence has an important impact on yield, by maintaining photosynthetic leaf area especially during the reproductive stage [1, 4–7]. With this in mind, researchers have extensively studied senescence regulation at the transcriptional level in model species such as *A. thaliana* [8–10] and, to a lesser extent, rice [11], tobacco [12], wheat [13], maize [14], barley [15] and sunflower [16–19].

NAC are plant specific TFs originally characterized in a Petunia NAM mutant [20] and subsequently in Arabidopsis CUC [21] and ATAF mutants. Among these proteins, ORE1 is one of the most studied NAC TFs in Arabidopsis leaf senescence. This TF is expressed during ethylene-induced senescence under the control of ETHYLENE INSENSITIVE 2, EIN2 [22]. ORE1 accelerates chlorophyll loss by directly activating the transcription of chlorophyll catabolic genes [23]. Consequently, ORE1 induces distinctive senescence features, including the degradation of nucleic acid, proteins and nitrogen recycling as well as the promotion of sugar transport [24, 25].

The structure of NAC proteins consists of two parts: the NAC domain (InterPro IPR003441) in the N-terminal region and the transcription regulatory regions (TRRs) in the C-terminal region. The NAC domain is subdivided in five well-conserved subdomains (A-E) and is responsible for dimerization and DNA binding, whereas TRR, which varies in sequence and length, behaves as a transcription activator or repressor [26].

NAC TF is a large gene family and was found in more than 160 plant species [27]. The evolutionary analyses of whole-genome duplication and the consequent clustering in orthologous genes are useful tools for inferring the functionality of NAC genes regarding complex and non-model species such as sunflower [26, 28, 29]. For example, Cenci et al. [28] developed an Orthologous Groups (OGs) framework determined by an expert sequence comparison of NAC genes from four species, two monocots (*O. sativa* and *Musa acuminata*) and two dicots (*V. vinifera* and *A. thaliana*) [28]. The members of each OG may derive from the same ancestor genes existing before the divergence of monocots and dicots and possibly share the same function. This classification is a reference for all monocot and dicot species and facilitates the identification of the most interesting NAC genes in species with scarce or null functional information.

The main goal of this study was to identify sunflower NAC genes (*HaNAC*) in the new version (HanXRQr2, 2020) of the sunflower genome recently published [30] and explore their evolutionary relationships by using the OGs framework in order to find HaNAC associated with leaf senescence in sunflower.

## Results

### Orthologous groups of NAC genes in *H. annuus* genome

The genomic analysis showed that the InterPro protein domain IPR003441 was present in 164 out of 71,289 automatically predicted proteins in the *H. annuus* genome V2. Additional genomic regions containing the domain IPR003441 were also detected and analyzed in this study. The sequences were manually curated to improve structural annotation, when necessary. Our analysis resulted in 151 potentially functional NAC genes (Additional file 1) and 15 pseudogenes/remnants.

A BLASTp analysis on whole NAC sequences set of four species representative of monocotyledonous (*M. acuminata, O. sativa*) and dicotyledonous species (*A. thaliana*, *V. vinifera*) assigned the 151 potentially functional NAC genes into 38 of 40 orthologous groups (OGs) defined by Cenci et al. [28] (Table 1). No gene could be assigned to OG-NAC-2c or OG-NAC-4 f. Although some genes remained without unequivocal assignation to any OG, 11 of these genes were close to the group of genes assigned to OGs 3. Six additional NAC genes, with divergent sequences, also remained unassigned to any OG. Two of these genes were tentatively grouped on the OG-NAC-Y group, whereas the other four genes were grouped in the OG-NAC-X.

**Table 1:** Number of HaNAC genes assigned to the Orthologous Groups

Orthologous group	a	b	c	d	e	f	g	h	
OG-1	3	2	1	4	1	1	3	6	
OG-2	8	3		2	2	6			
OG-3	4	4	2	5	6				11
OG-4	3	2	2	7	4		2		
OG-5	10	3	1						
OG-6	2	3	2						
OG-7	2	4	1	4	2	8			
OG-8	3	6							
OG-X									4
OG-Y									2

**Fig. 1:**
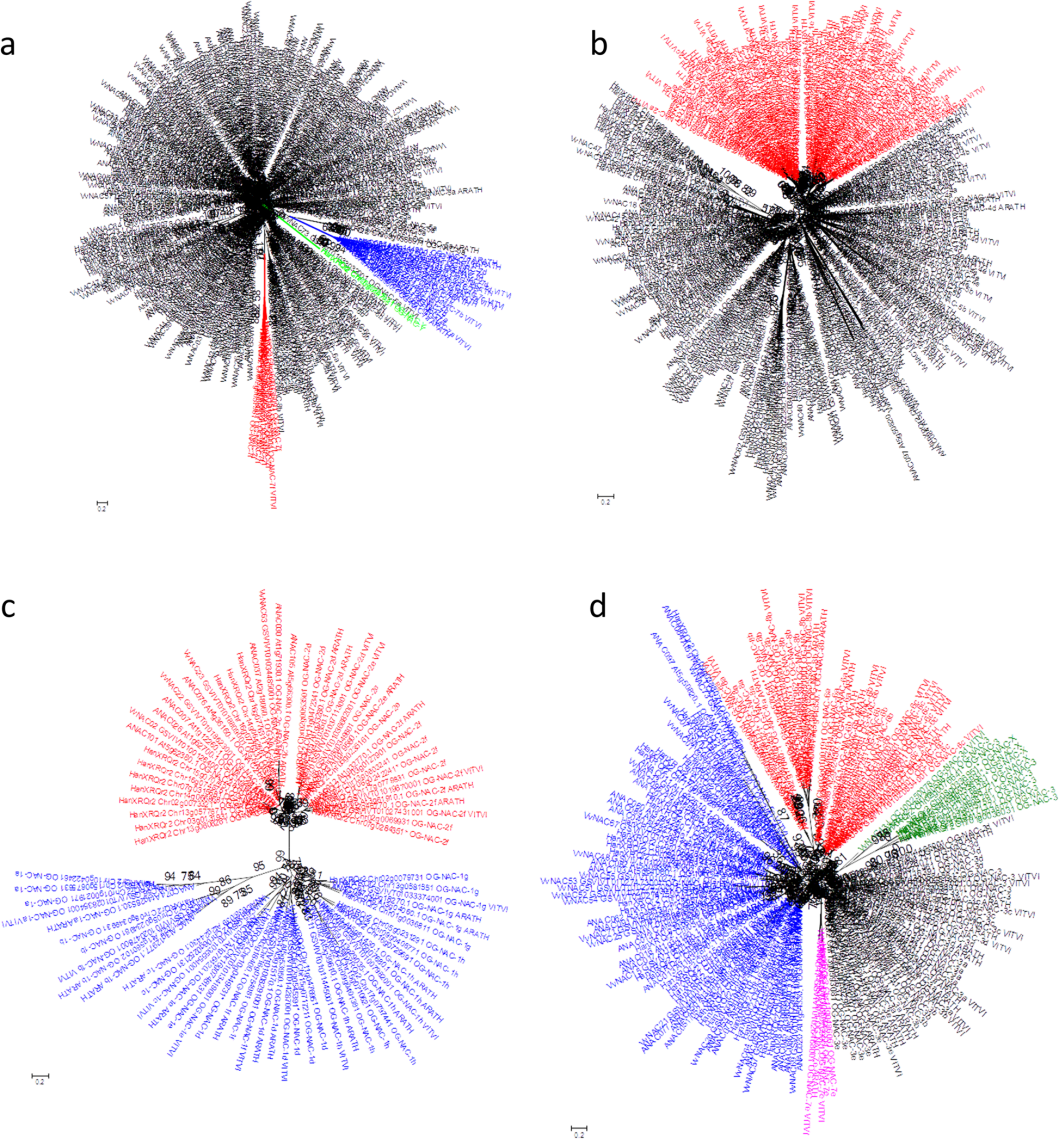
Phylogenetic analysis of OG of HaNAC genes. **a** Red: OG-NAC-7f, blue: OGs-NAC-7a, 7b, 7c and 7d, green: OG-NAC-Y. **b** Red: OG-NAC-1 and OG-NAC-2. **c** red OG-NAC-2, blue: OG-NAC-1. **d** Fuchsia OG-NAC-7e, green OG-NAC-3 (unique for sunflower), red OGs-NAC-6 and -8, blue OGs-NAC-4 and -5

A phylogenetic tree of the 305 NAC protein sequences of *H. annuus*, *V. vinifera* and *A. thaliana* was built based on 88 positions retained after alignment cleaning (Additional files 2, 3, 4, 5, 6). Three clusters with very high support (aLRT = 1) were connected to the remaining tree by long branches. One of these three clusters (in red, Fig. 1a) included all the sequences (8 of *H. annuus* and 1 of *V. vinifera*) assigned to the OG-NAC-7 f. Another cluster (in blue; Fig. 1a) contained all the sequences (11 of *H. annuus*, 7 of *A. thaliana* and 5 of *V. vinifera*) assigned to the OGs-NAC-7a, 7b, 7c and 7d. The third cluster (in green; Fig. 1a) included the two *H. annuus* NAC sequences assigned to OG-NAC-Y. Another phylogenetic tree built with 181 aligned positions of the 23 sequences in the blue cluster. Four OG-specific clusters contained all the analyzed sequences, with aLRT support ranging between 0.87 and 1 (Additional files 2, 3, 4, 5, 6).

After removing the 34 above mentioned diverging sequences, we performed the phylogenetic analysis with the remaining sequences (271). The new phylogenetic tree, which was based on 94 aligned positions, resulted in clusters consistent with most of the OG assignations (Additional files 2, 3, 4, 5, 6); the phylogenetic information in the analyzed alignment, however, was insufficient to resolve some of the OGs in specific clusters.

To gain insight in the NAC sequence phylogeny, we divided the tree in two main clusters, one containing the sequences assigned to OGs-NAC-1 and 2 (in red; Fig. 1b) and another with the remaining sequences. New rounds of phylogenetic analysis were performed to construct trees with the sequences of the two clusters.

The tree containing the 86 sequences from OGs-NAC-1 and -2 (125 aligned positions) was subdivided in two very well-supported clusters (aLRT = 1), one containing the sequences assigned to OGs-NAC-1 and the other to OGs-NAC-2 (Supplementary material 2–6; Fig. 1c). Two phylogenetic trees built for OGs-NAC-1 (131 positions in 44 sequences) and OGs-NAC-2 (158 positions in 43 sequences) led to OG assignments that were consistent with the sequence clustering, even if some OG specific clusters had low aLRT support (Additional files 2, 3, 4, 5, 6). The clusters including sequences assigned to each OG-NAC-1 h and OG-NAC-2a could be split into two sub-clusters containing at least a sequence from each considered species.

The analysis of the 185 sequences included in the largest cluster (in black; Fig. 1b) and based on 92 aligned positions allowed the identification of two small and three large clusters (Additional files 2, 3, 4, 5, 6; Fig. 1d). The cluster represented in fuchsia (aLRT = 0.99) contained all the sequences assigned to OG-NAC-7e, whereas the cluster shown in green (aLRT = 0.97) contained the *V. vitis* sequence VvNAC43 (previously assigned to OG-NAC-3d) and 12 *H. annuus* sequences, which had remained unassigned to any OG with the previous analysis.

The cluster in red contained all the sequences assigned to OGs-NAC-6 and -8, whereas the one indicated in blue consisted of all the sequences assigned to OGs-NAC-4 and -5. The one in black, on the other hand, contained the sequences assigned to OGs-NAC-3 as well as three *H. annuus* non-assigned sequences (OG-NAC-X).

A phylogenetic tree was built using the NAC sequences on each of the three clusters containing sequences assigned to more than one OG. The tree built with the 39 sequences assigned to OGs-NAC-6 and -8 was based on 119 aligned positions. Four out of five clusters corresponding to the five OGs had an aLRT support higher or equal to 0.9, whereas the cluster containing all the sequences assigned to the OG-NAC-6b displayed very low support (Additional file 4). The tree built with the 82 sequences (124 positions) assigned to the OGs-NAC-4 and -5 contained ten clusters consistent with an aLRT support higher or equal to 0.88 (Additional file 5).

The tree built with the 46 sequences (132 positions), which were mainly assigned to the OGs-NAC-3, consisted of six main clusters (Additional file 6). Each of five of them contained all the sequences assigned to OG-NAC-3a, 3b, 3c, 3d and 3e. The sixth cluster contained two *V. vinifera* sequences, previously unassigned to a specific OG but considered close to the OGs-NAC-3 (VvNAC42 and VvNAC45), and three unassigned *H. annuus* NAC genes.

### Chromosomal location of HaNAC genes

In this study, we localized the eight HaNAC genes previously described by our group (HaNAC001, HaNAC002, HaNAC003, HaNAC004, HaNAC005, HaNAC006, HaNAC007 and HaNAC008) in the latest version of the genome (Additional file 1) [31]. The criterion for identifying the remaining 138 HaNAC genes consisted of numbering these genes successively according to their position in the genome. The first gene annotated in the genome was HaNAC009 and the last one was HaNAC0146. Five other genes that had not been previously annotated into the genome were named as HaNAC147, HaNAC148, HaNAC149, HaNAC150, HaNAC151, and subsequently localized in the genome.

**Fig. 2:**
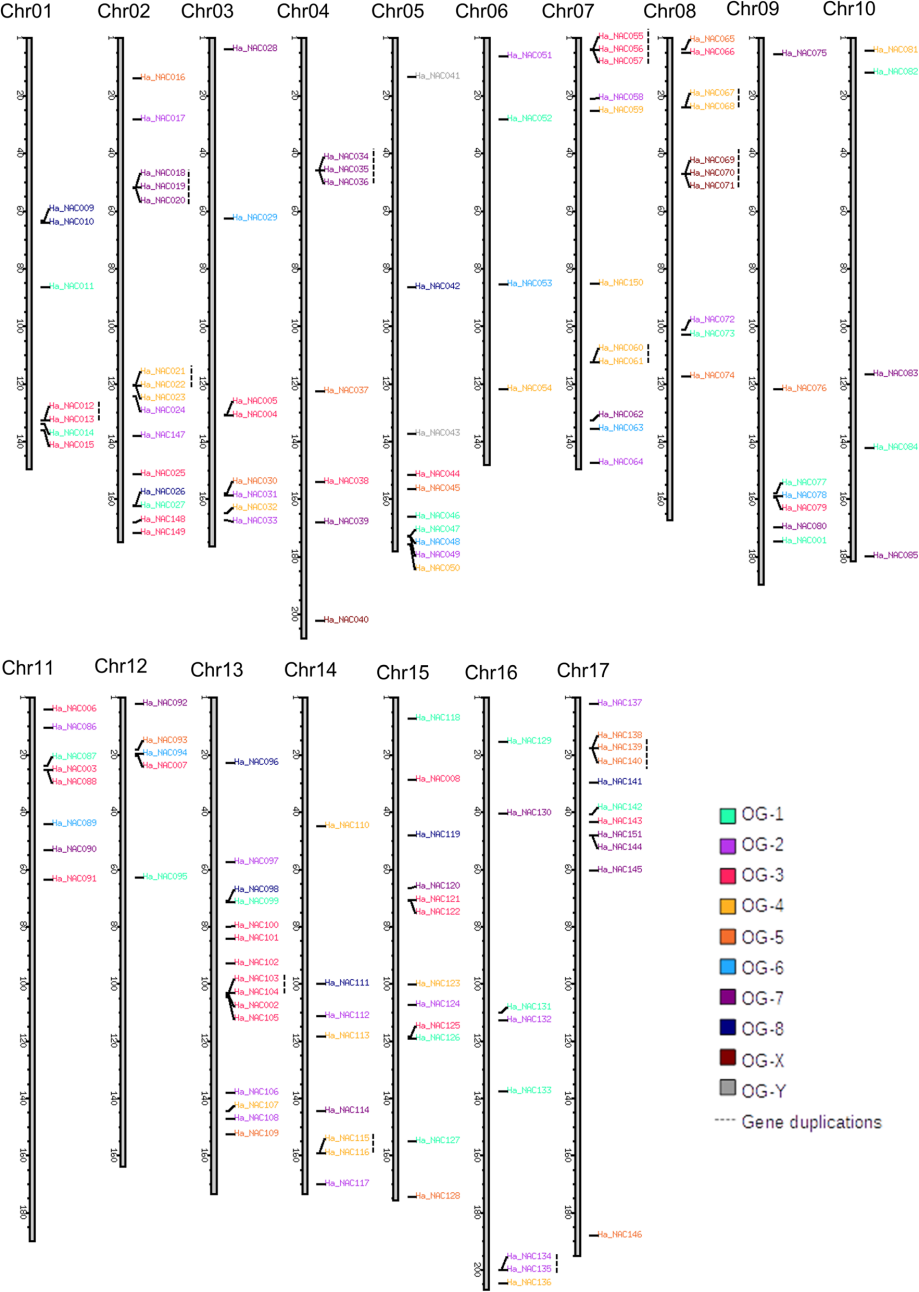
Chromosomal location of NAC TF genes in sunflower. A graphical representation of putative HaNAC genes location on each sunflower chromosome. Each family of OG is distinguished with a different color according to the figure reference. The dashed lines on the gene groups, on the right, indicate tandem duplicated genes

A schema of the localization of the NAC genes into the sunflower genome by using the KaryoploteR package in R revealed an apparent random OG distribution of HaNAC genes in each of the 17 sunflower chromosomes (Fig. 2). Chromosomes 2 and 13 contained the largest number of HaNAC genes (15 each; 10% of total genes). Chromosome 6, on the other hand, contained the minimum (four; 2.7%). Chromosomes 17, 11 and 5 contained detectable clusters but no chromosome region enrichment. Both p and q chromosome arms contained genes close to the centromeric or telomeric regions.

### NAC member duplications in *H. annuus*

*H. annuus* genome contains traces of multiple rounds of genome multiplication: the most recent, a whole genome duplication (WGD) specific to *Helianthus* spp.: a whole genome triplication (WGT), specific of the Asterids II clade [30] and a more ancient γ-WGT, which is shared between asterids and rosids [32]. According to our analysis, there were NAC genes originated by polyploidization events and tandem duplications. Eighteen couples of NAC genes occurred in regions duplicated by the last *H. annuus* WGD detected in the first version of its genome sequence [30] (Table 2).

**Table 2:** HaNAC paralogs originating from the Whole Genome Duplication specific of *Helianthus* spp.

Orthologous groups	Paralogs	
OG-NAC-1a	Ha_NAC082	Ha_NAC118
OG-NAC-1b	Ha_NAC073	Ha_NAC047
OG-NAC-1 g	Ha_NAC099	Ha_NAC027
OG-NAC-2a	Ha_NAC064	Ha_NAC117
OG-NAC-2a	Ha_NAC108	Ha_NAC033
OG-NAC-2e	Ha_NAC049	Ha_NAC072
OG-NAC-2f	Ha_NAC051	Ha_NAC137
OG-NAC-2f	Ha_NAC106	Ha_NAC031
OG-NAC-3	Ha_NAC025	Ha_NAC101
OG-NAC-3a	Ha_NAC008	Ha_NAC143
OG-NAC-3c	Ha_NAC066	Ha_NAC007
OG-NAC-4c	Ha_NAC107	Ha_NAC032
OG-NAC-5a	Ha_NAC074	Ha_NAC045
OG-NAC-6b	Ha_NAC094	Ha_NAC094
OG-NAC-7b	Ha_NAC075	Ha_NAC028
OG-NAC-7b	Ha_NAC085	Ha_NAC092
OG-NAC-7d	Ha_NAC062	Ha_NAC114
OG-NAC-7f	Ha_NAC080	Ha_NAC130
OG-NAC-8a	Ha_NAC119	Ha_NAC141
OG-NAC-8b	Ha_NAC098	Ha_NAC026

Genes of the same OG and mapped at a distance lower or equal to 100 kb were considered tandem duplications. The analysis retrieved 30 genes organized in 12 different tandems containing two or three genes (Fig. 2).

### Exon/intron structure analysis

The basic structure of the NAC gene is composed of three exons. The first exon contains A and B subdomains [33] and ends at the first nucleotide of the first codon after the B subdomain; the second exon contains the C and D subdomains and ends at the third nucleotide of a codon; the third exon begins with the E subdomain and contains all the C-terminal region of the gene that includes the TRR [28]. The basic structure of three exons was found in the genes assigned to OGs 1, 2, 3, 6 and 8 as well as for the OG-NAC-4b, 5a and 7e. The unique exception was HaNAC146, whose first two exons were merged. The genes assigned to the OG-NAC-7a had also three exons; however, they corresponded to exons 1 and 2 of the basic structure, respectively. OG-NAC-7b showed a similar composition. In the OG-NAC-7c and -7d sequences, which have an additional exon containing few codons at the beginning of the coding sequence, the third and fourth exons corresponded to exons 1 and 2 of the basic structure. The sequences of OG-NAC-7a, −7b, 7c and -7d, in addition to the C and D subdomain, the third exon contained, also the E subdomain. This finding suggests an exon merge in the common lineage of these four OGs. As in other species [28], sequences included into OG-NAC-7b, −7c and -7d had additional exons in the C-terminal region containing the highly variable transcription regulatory regions (TRRs). Similarly, in most of the sequences assigned to OGs 4, 5 and OG-NAC-7e, a variable number of exons were also present in the C-terminal, in addition to the three exons of the basic structure (Additional file 7a and b).

### Structural analysis of NAC proteins

NAC proteins had a characteristic NAC domain in the N-terminal with the capacity to interact with the CGT[AG] core sequence in target genes. Despite the conserved NAC domain in the NAC TF family, the members of this family presented variability within the group. For example, some NAC genes had a variable number of NAC domains (named chimeric NAC domains), whereas others had extra domains, such as transmembrane domains [26].

According to a protein sequence analysis by Pfam, NAC domains of all the analyzed genes were of approximately 126 amino acids, except for HaNAC091 (26 amino acids), and all located in the N-terminal of the protein (Additional file 1). All the analyzed proteins had a unique NAC domain, although in some cases they contained other extra domains. Some OGs were characterized for these extra domains. For example, in the N-terminal, four of five genes of the OG-X had a Claterim_lim domain, whereas OG-7a had a CpXC domain. On the other hand, OG-3 had a Ring-hydroxyl-B domain in the C-terminal.

Additionally, according to a transmembrane domain analysis, HaNAC101 and HaNAC111 contained transmembrane domains at the N-terminal, whereas HaNAC061, HaNAC067, HaNAC050, HaNAC82 (all within the OG-4 subfamily) and HaNAC110 presented these domains at the C-terminal (Additional file 1).

**Fig. 3:**
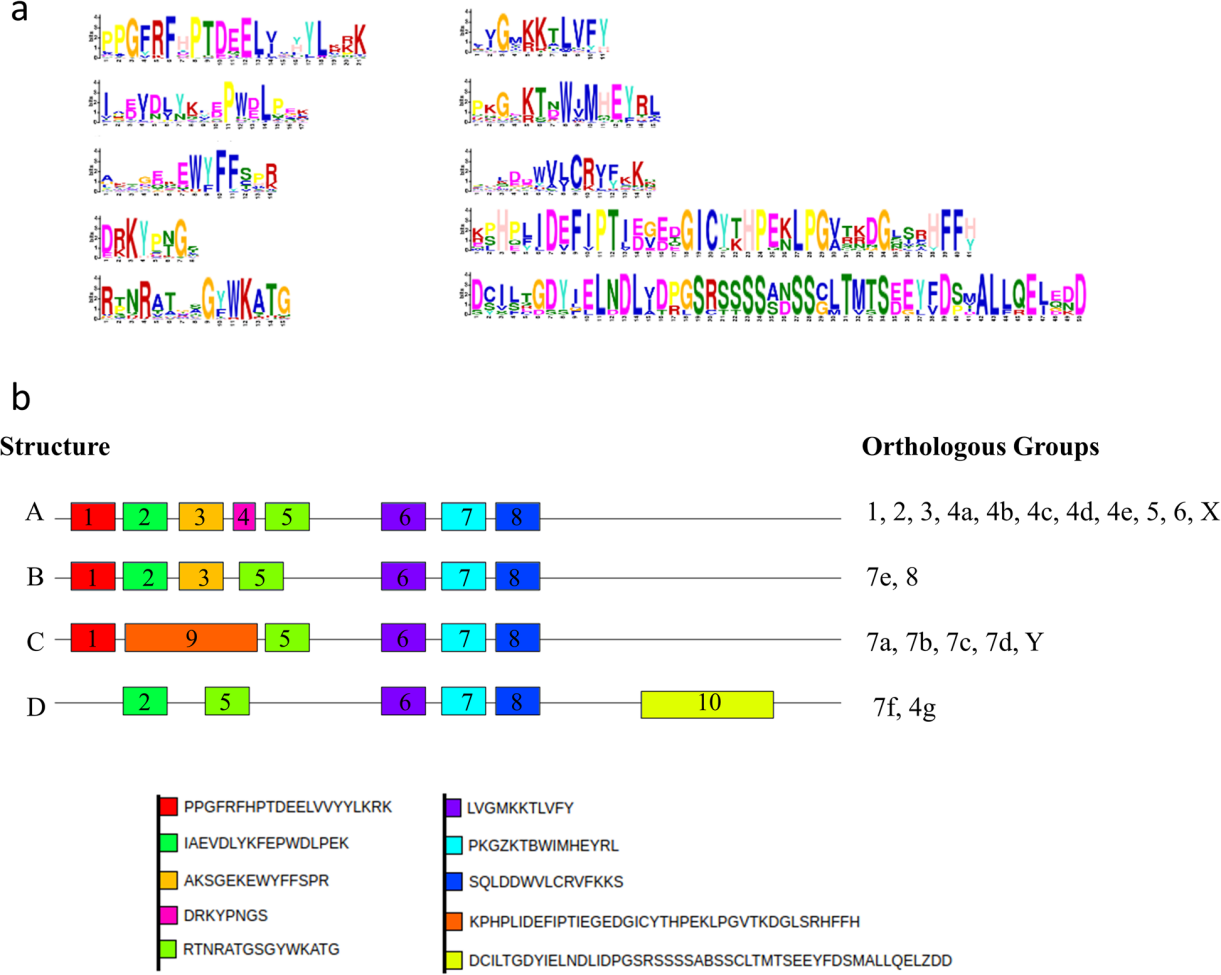
MEME protein analysis. **a** MEME motifs in the 151 HaNAC proteins. **b** Four structures (A, B, C, D) with different motifs. Motifs are displayed in different colored boxes and sequence information is provided for each motif. The letters Z and B refer to equal possibility of the amino acids Q/E or N/D, respectively

Using MEME and LOGO software we evaluated the common motifs in the HaNAC proteins. Here we identify ten different motifs, of which four (motif 5, 6, 7, 8) occurred in almost all sequences (Fig. 3a) [34]. Motifs 1–7 and 9 were located inside the NAC domain in the N-terminal. The motifs were organized in different structures, named A, B, C and D (Fig. 3b). The distance between the motifs was variable among the HaNAC proteins. However, motifs 2, 3, 4, 5 had a tendency to cluster in structure A (reading from left to right).

Almost all NAC genes of OGs 1, 2, 3, 4a, 4b, 4c, 4d, 5, 6 and X contained the structure A conformed by eight motifs [1–8]. Some exceptions showed several motif deletions: motifs in HaNAC016, HaNAC045, HaNAC076, all of the OG-5a group and HaNAC91 from the OG-3. This last gene had two exons, whereas the rest of their OG had 3 exons.

OG-7e belonged to structure B, whose structure was highly similar to structure A, but without motif 4. Other OG belonging to structure B were OGs-8 and the proteins HaNAC135 and HaNAC021 (of the OG-2b and OG-4e, respectively). In accordance with the variability in exon numbers, OGs-7 contained variable types of structures. OG-7a, OG-7b, OG-7c and OG-7d belonged to structure C and had an unequal motif, the motif 9. This motif may be functionally redundant to motifs 2, 3, 4 and 5.. Finally, OG-7f, which belonged to structure D, showed a different motif that may be associated with the TRR domain.

All the motifs positioned on the NAC domain were similar to the motifs in *Arabidopsis thaliana* NAC proteins. According to a comparative analysis between the motifs found here and the subdomains reported in Kikuchi et al. [33] and Puranik et al. [26], motifs 1 and 2 corresponded to the subdomain A and subdomain B, respectively, whereas motifs 3, 4, and 5 corresponded to the subdomain C. In addition, motifs 6 and 7 corresponded to subdomain D, whereas motif 8 corresponded to subdomain E. Motif 10 on the structure D was in the C-terminal and showed no similarity with *Arabidopsis thaliana* NAC proteins.

### Regulatory sequences of NAC genes

**Fig. 4:**
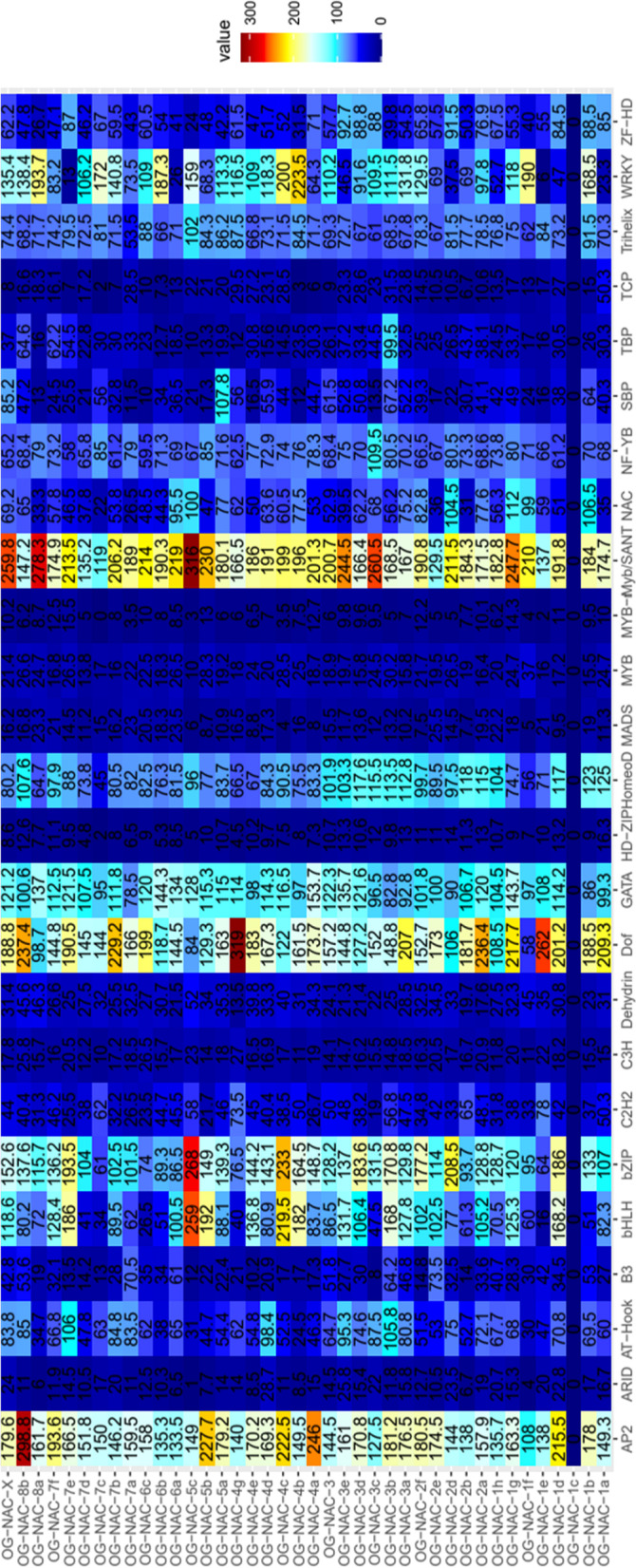
Regulatory sequences in each Orthologous Group (OG). Mean number of times that a certain TFBS appeared in the sequence of each gene was calculated for the analysis. A total of 2000 bp upstream of ATG was evaluated for a different Transcription Factor Binding Site (TFBS) in each OG

TFs regulate cell processes by binding a specific (TFBS) DNA motif on promoter regions and, therefore, affect downstream gene expression. However, not all members of a TF family have the same functions or have the same expression profile in all the tissues or in all developmental stages. In this study, we evaluated the differences in the promoter region of the HaNAC using the database PlantPAN 3.0 [35]. The analysis carried out for each OG showed the mean value of TFBS frequency from different TF families (Fig. 4).

Most representative TFBS families along the 151 HaNAC were AP2, ARID, AT-Hook, B3, bHLH, bZIP, C2H2, C3H, Dehydrin, Dof, GATA, HD-ZIP, Homeodomain, MADS, MYB, MYB-related, Myb/SANT, NAC, NF-YB, SBP, TBP, TCP, Trihelix, WRKY and ZF-HD.

Some OGs were defined by specific TFBS families. For example, AP2 TFBS was over-represented in OG-8b and moderately high in OG-4a, OG-4c and OG-5b. At-hook TFBS were over-represented in OG-3b, OG-4d and OG-7e, whereas bHLH and bZIP TFBS were particularly high in OG-5c. Dof TFBS were over-represented in OG-4 g and OG-1e, whereas Myb-SANT TFBS were over-represented in OG-X, OG-8a, OG-3c but even more in OG-5c. NFYB, SBP and TBP TFBS were particularly high in OG-3c, OG-5a and OG-3b, respectively.

The heatmap plot showed a unique TFBS pattern for each OGs. In this pattern, some TFBS were more important than others in discriminating between OGs. OG-NAC-1a and OG-NAC-1c had similar TFBS values for all the analyzed TFs, except for NAC and WRKY TFBS. In addition, OG-NAC-1d, − 1e, − 1f, −1 g and 1 h had similar TFBS patterns, except for AP2, bHLH, Dof, Myb/SANT, NAC and WRKY TFBS. Moreover, the OGs of subfamily 2 had different values for Dof, NAC, WRKY and bZIP TFBS. Other HaNAC subfamilies showed similar results.

### Expression of NAC genes profiles during senescence in cultivated sunflower

NAC TF family regulates several physiological changes during plant development. The senescence is the last stage in plant cycle life and starts after the flowering period (anthesis) and lasts during the grain filling until the complete dehydration of the plant and seeds. This process is a complex mechanism that accompanies the molecular recycling of carbon and nitrogen from leaves to seeds.

**Fig. 5:**
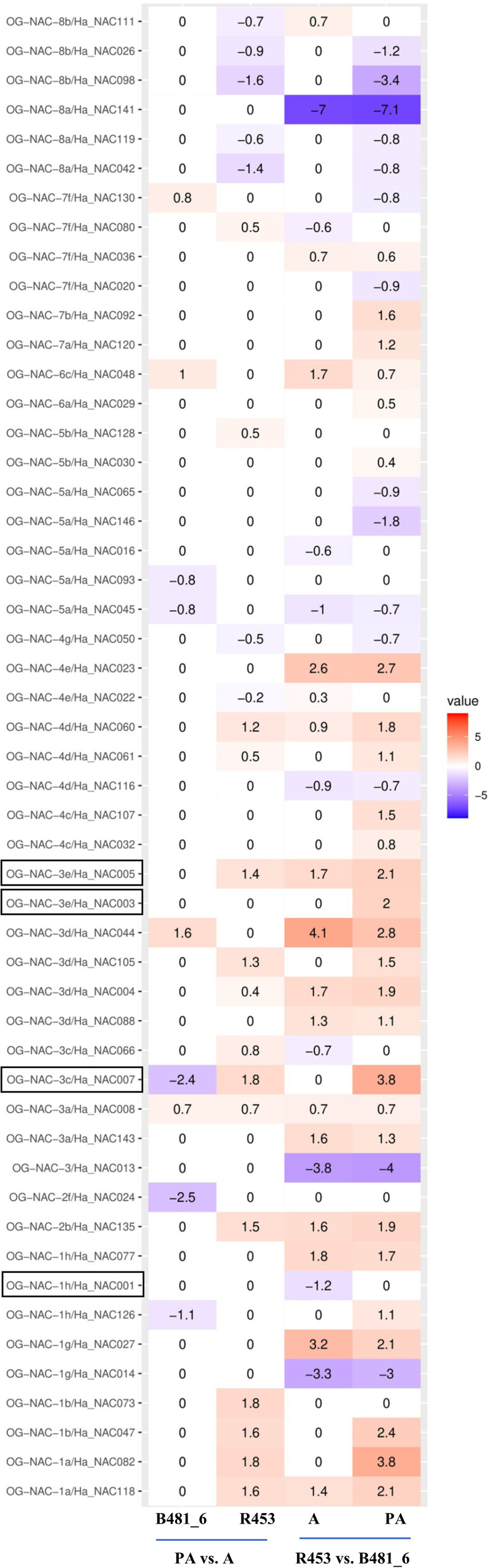
Heatmap of gene expression values for HaNAC genes in two sunflower contrasting lines for senescence. The expression ratio from four analyses are presented: post-anthesis (PA) vs anthesis (A) in the R453 line (early senescence), post-anthesis (PA) vs anthesis (A) in the B481–6 line (delayed senescence), R453 line vs B481–6 line at anthesis (A), and R453 vs B481–6 at post-anthesis (PA). Color intensity corresponds to the expression ratio at logarithmic scale (log2) values with a *p*-value lower than 0.05. Red: upregulated, blue: downregulated

To evaluate the functionality of the HaNAC associated with senescence in sunflower, we performed an RNAseq experiment of two contrasting sunflower lines in time-to-leaf senescence traits. Genotype R453 showed an early senescence phenotype, whereas B481–6 had a delayed senescence phenotype. The analysis consisted of two evaluation times, at anthesis (A) and post-anthesis (PA), and four comparisons: line B481–6 post-anthesis vs. anthesis, line R453 post-anthesis vs. anthesis, R453 vs B481–6 at anthesis, R453 vs B481–6 PA post-anthesis (Fig. 5, Additional file 8).

Differentially expressed genes between post anthesis vs. anthesis were associated with molecular changes during leaf senescence. R453 (early senescence phenotype) displayed a singular expression profile. For example, genes of OG-1a (*HaNAC118, HaNAC082*), OG-1b (paralogues: *HaNAC047* and *HaNAC073*) and OG-2b (*HaNAC135*) had log_2_ ratio values above or equal to 1.5, whereas a gene of OG-8b (*HaNAC098*) had log_2_ ratio values below or equal to 1.5. These HaNAC genes were not differentially expressed in B481–6 (delayed senescence phenotype). Conversely, B481–6 had only one gene of OG-3d with log_2_ ratio value above 1.5 (*HaNAC044*) and two genes of OG-3c and OG-2f (*HaNAC007* and *HaNAC024*, respectively) with log_2_ ratio values below 1.5. *HaNAC007* was an interesting gene because of its contrasting expression depending on the genotype: upregulation in R453 and downregulated in genotype B481–6.

Any difference between gene expression patterns of the two contrasting lines in a given time, could also be useful for assessing the senescence process. Indeed, differences in the observed phenotype could be explained by a different expression pattern of a given gene during anthesis (A) or post-anthesis (PA). For example, *HaNAC014, HaNAC013* and *HaNAC141* displayed downregulated patterns in R453 in comparison to B481–6 (during anthesis and post-anthesis), whereas *HaNAC023* and *HaNAC027* showed an upregulated expression (during anthesis and post-anthesis). These genes were only differentially expressed between genotypes and not in the PA/A comparison.

Other genes appear to be involved in the senescence process depending on both time and genotype. During post-anthesis, *HaNAC082*, *HaNAC047* and *HaNAC007* displayed an upregulated profile, while *HaNAC146* and *HaNAC98* showed a down regulated profile, in R453. In this line, on the other hand, *HaNAC044* was upregulated during anthesis.

### Phenotyping assay

Orthologous relationships are useful for inferring gene functionality. We analyzed Gene Ontology for all the *Arabidopsis thaliana* OGs described by Cenci et al. [28] (information provided by TAIR database) enriched in the terms “leaf senescence” and “programmed cell death” (Additional file 9). We found that *Arabidopsis thaliana* genes belonging to OG-1 h, OG-3e and OG-3c had ontology terms associated with leaf senescence. For that reason, we selected four genes belonging to OG-1 h, OG-3e and OG-3c: HaNAC001, HaNAC003, HaNAC005 and HaNAC007, for functional validation of HaNAC [18]. These genes also showed differential expression in RNAseq assay (Fig. 5).

We generated *Arabidopsis thaliana* transgenics lines for these four sunflower genes and evaluated their phenotype. Two independent lines of *Arabidopsis thaliana* were selected for each gene construct. According to a qPCR analysis, the transgenic lines showed different transgene expression levels (Additional file 10). Growth and morpho-physiological traits of the eight lines (HaNAC01–1, HaNAC01–4, HaNAC03–3, HaNAC04–5, HaNAC05–1, HaNAC05–5, HaNAC07–2, HaNAC07–5) were quantified in the automated phenotyping platform PHENOPSIS.

**Fig. 6:**
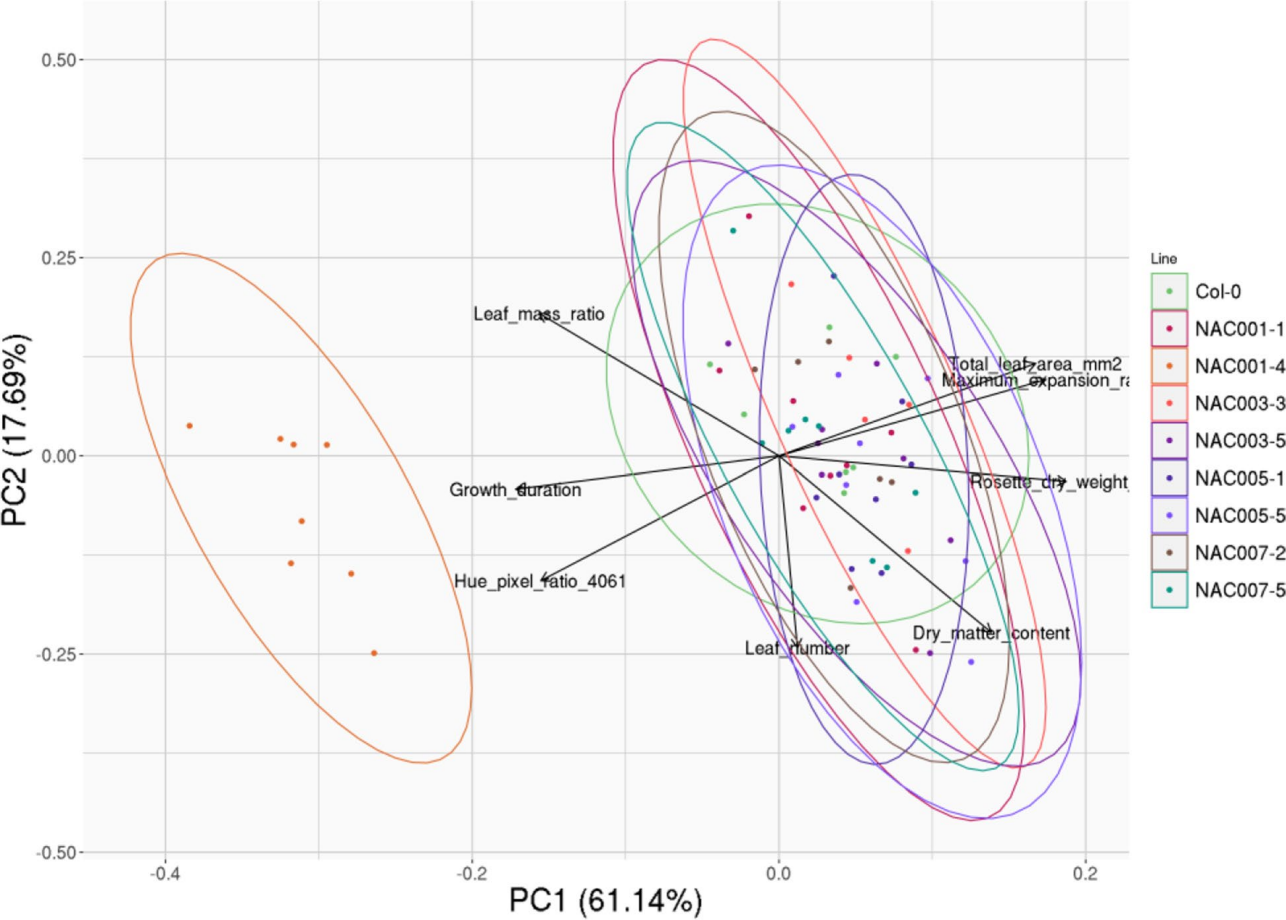
PCA analysis for eight independent overexpressing *Arabidopsis thaliana* lines and Col-0. Each color represents a different independent line. Orientation and distance from center of gravity indicates association of the projected variables with PC1 and PC2 (78.6% of variance)

The two first components of a PCA analysis performed with leaf number, rosette dry weight, total leaf area, Hue pixel ratio (40–61), dry matter content, leaf mass area, growth duration and maximum expansion rate as active variables explained 78.6% of the total variability of the measured data. The figure shows that the morphophysiological traits growth duration and rosette dry weigh contributed primarily to the PC1 axis, meanwhile dry matter content and the leaf number contributed primarily to the PC2 axis. Projection of individual observations revealed that individuals from the line HaNAC01–4 were grouped in a single contrasted cluster in comparison with the other lines and the control Col-0. HaNAC01–4 cluster differs from the others mainly by a shift on the x-axis, showing that this line had more growth duration and less rosette dry weigh that the other ones. HaNAC01–4 was the line with highest expression levels of the transgene HaNAC001 (Fig. 6).

**Fig. 7:**
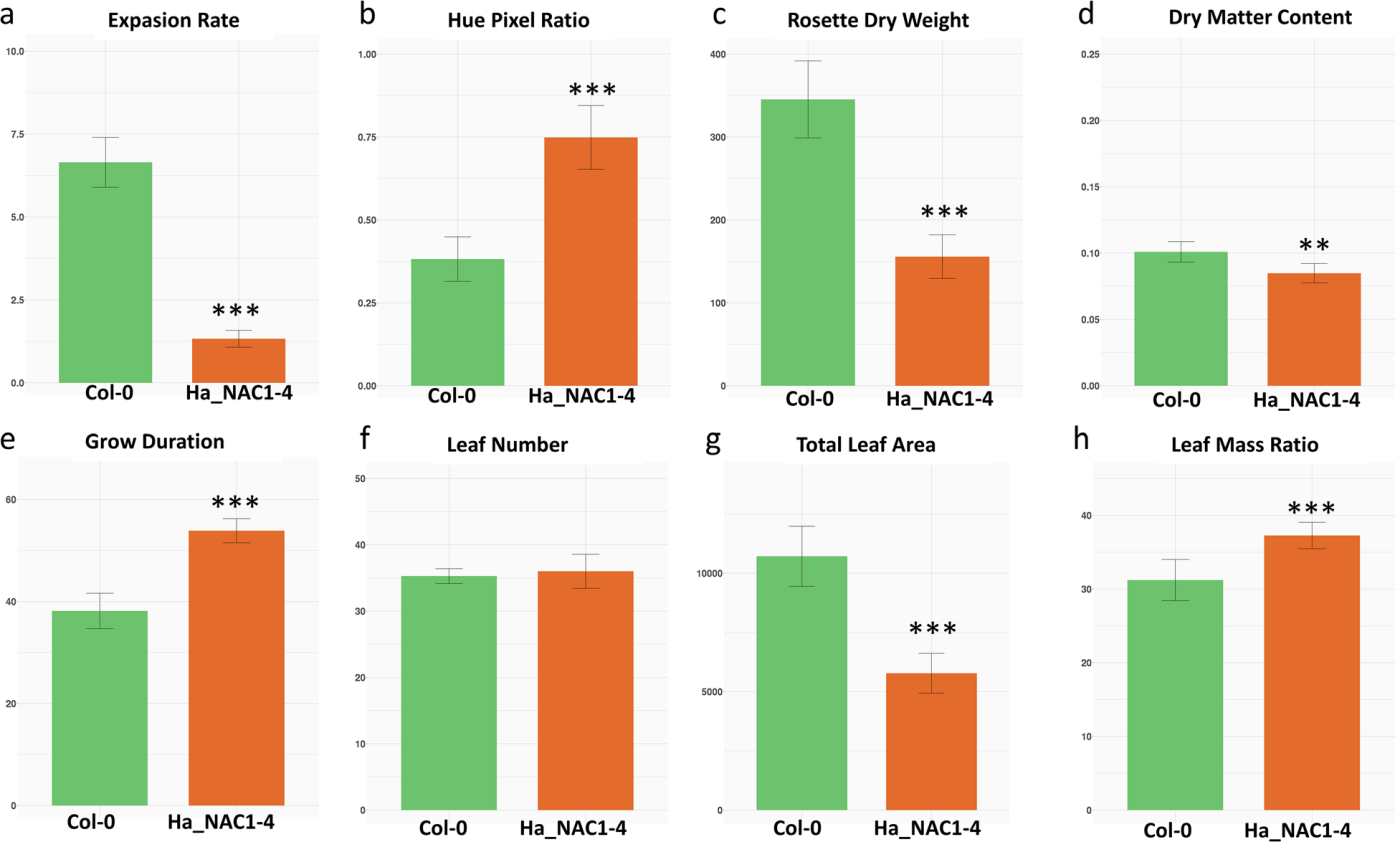
Morphophysiological traits of Col-0 and HaNAC001–4. All the traits were analyzed by ANOVA and Tukey post-hoc test (*** < 0.001, ** < 0.01). **a** Maximum expansion rate (mm^2^ d^− 1^), **b** Hue pixel ratio (40–61), **c** Rosette dry weight (mg), **d** Dry matter content, **e** Growth duration (days), **f** Leaf number, **g** Total leaf area (mm^2^), **h** Leaf mass ratio (mg mm^− 2^)

**Fig. 8:**
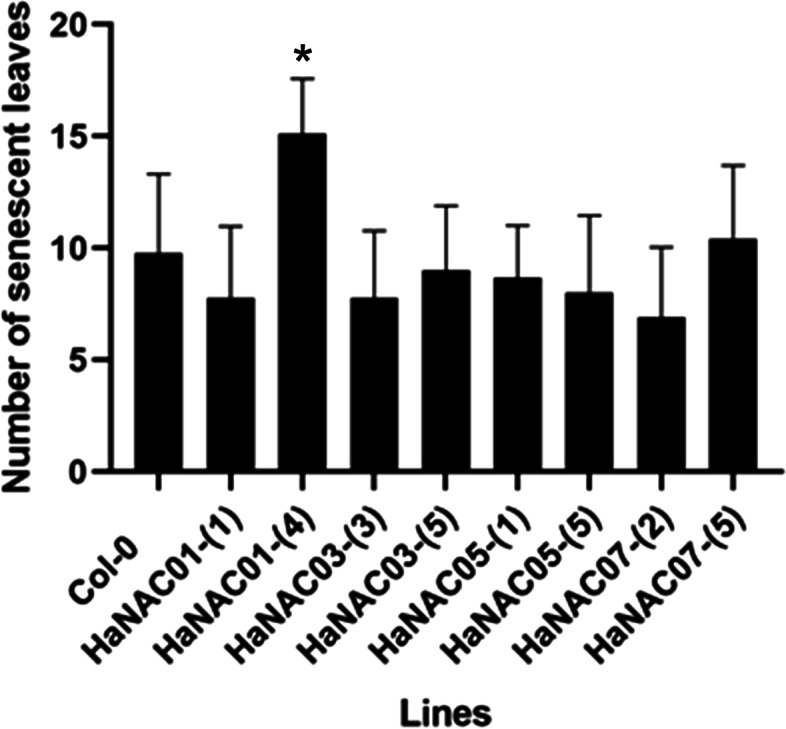
Number of senescent leaves. The number of senescing leaves of Arabidopsis transgenic lines HaNAC01–1, HaNAC01–4, HaNAC03–3, HaNAC04–5, HaNAC05–1, HaNAC05–5, HaNAC07–2, HaNAC07–5, and the wild-type Col-0 were measured at flowering stage. The number of senescent leaves were analyzed by ANOVA and Tukey post-hoc test (* < 0.05)

Compared with *Arabidopsis thaliana* Col-0, the HaNAC01–4 line showed a similar number of leaves but significantly lower dry weights and leaf area values (Fig. 7). This was associated with a lower growth rate and growth duration in the transgenic line compared to the wild-type (Fig. 7). The mean Hue pixel ratio (40–61), i.e. the yellow and light green proportion of pixels in all the leaves of each plant, of HaNAC01–4 line plants was twice the ratio of Col-0 plants in concordance with the number of senescing leaves at flowering (Fig. 8).

## Discussion

NAC transcription factors are a large family of genes specific to plants and involved in several functions: developmental programs, senescence, formation of secondary walls, and biotic and abiotic stress responses [26]. Despite high sequence similarity in Arabidopsis, NAC genes regulate senescence by different molecular mechanisms and under different stimuli (age, abiotic or biotic stress) [8].

A proper comparison of NAC gene sets of different species and functional transfer (i.e. prediction of the most probable function of uncharacterized NAC genes) requires clarifying phylogenetic relationships among them. In fact, the closer the phylogenetic relationships, the higher the probability of function conservation.

Particularly, a classification based on orthogroups (OGs) was proposed for large gene families [28, 36] to facilitate interspecific comparisons. An OG includes all the gene family members that originated from a common ancestor gene existing before the radiation of the considered species.

Phylogeny analyses of NAC family genes are challenging because of the presence of the C terminal high variable region (TRR) and the high number of members within this family. In this study, we used an iterative strategy. A phylogenetic tree was obtained with the whole set of NAC genes and sequences in sharply divergent clusters were separated and submitted to a new analysis round, in which the reduced divergence among sequences improved the alignment quality and increased the number of positions retained for subsequent phylogenetic reconstruction. This approach allowed better resolution of the phylogenetic relationships between sequences.

Most analyzed *H. annuus* NAC genes (135/151) could be unequivocally assigned to 38 of the 40 previously established OGs by BLASTp analysis on NAC sequence database [28]. A tBLASTn analysis in *H. annuus* genome with *V. vinifera* genes assigned to these OG confirmed that *H. annuus* genome has no OG-NAC-2c and OG-NAC-4f member. Iterated phylogenetic analyses including whole NAC gene sets of *A. thaliana* and *V. vinifera* gave clusters highly consistent with the prediction of BLASTp assignation.

Among the 17 unassigned genes, two with close sequences (HaNAC041 and HaNAC042) were very divergent from all other NAC in the four species NAC sequences database. The BLASTp analysis in the global database indicates that similar (and possibly orthologous) sequences are present only in some dicot species. Among them, the *V. vinifera* VvNAC72 sequence (XP_002282899.1) was not considered by Cenci et al. [28]. The other 15 sequences were detectable in two supported clusters with *V. vinifera* sequences. Three of the remaining 15 unassigned sequences were in a cluster with two *V. vinifera* genes (VvNAC42 and VvNAC45), which could not be unambiguously assigned to any of the 40 OGs in previous analyses. The other 12 unassigned genes formed a cluster with a *V. vinifera* gene (VvNAC43) assigned to OG-NAC-3d, which is a probable pseudogene [28]. The 17 unassigned genes could be part of undescribed OGs. An analysis with more species is essential before drawing conclusions.

A total of 151 HaNAC proteins were then analyzed by searching for extra domains and motifs. We found ten motifs that allowed us to classify the HaNACs in four different structures. Almost all motifs belong to reported subdomains for NAC proteins. Even more, the sequence DEEL in motif 1, can be related to calcium-binding motifs present in the EF-hand of calcium-dependent protein kinases. This result suggests that HaNAC could be involved in signaling processes regulated by Ca^2+^, at least in OGs that had motif 1 [27]. According to a recently published report, conserved consensus sequences G-Y-W-K-A/T-T-G-x-D-× 1–2-I/V, G-x-K-K-x-L-V-F-Y, and T-x-W-x-M-H-E-Y based on the analysis of 160 plant species, could be found in motifs 5, 6, and 7 in HaNAC proteins [27]. The thorough analysis of these conserved consensus sequences supported the inference of functionalities with crystal structures [27]. In comparison with ANAC19 crystal structure, motif 5 could be responsible for the specific recognition of DNA and binds at the major DNA groove, whereas motif 6 and 7 could bind the backbone of the DNA [37].

Gene regulatory sequences could discriminate against the OGs and possibly regulate their functions in a tissue or time specific manner. We found TFBS for TF families like AP2, bHLH, bZIP, MYB and WRKY that were already reported in the regulation of NAC genes. But also, we found other TFs less known in the regulation of NAC expression like Dof TF [38]. Dof TF is apparently an unique Zn finger domain of plants described for the first time in *Zea mays* [39]. Dof TF are involved in the regulation of metabolism, phytohormone responses, photoperiodic regulation, hormonal regulation, and there is reported of interaction between this TF and genes belongs to bHLH and MYB TFs [38]. Even more, the subdomain D (motifs 6 and 7, in this analysis) have a hydrophobic L-V-F-Y sequence reported as a negative regulator of transcription for WRKY, Dof, and AP2. In this way the NAC and Dof TF could be involved in a negative feedback regulation network [40].

We explored the functional validation of four HaNAC genes related to senescence in sunflower. HaNAC001 (OG-1 h), HaNAC003 (OG-1e), HaNAC005 (OG-3e) and HaNAC007 (OG-3c) had orthologous members related to senescence in *Arabidopsis thaliana*, and presented interesting expression profiles in the RNAseq assay. An analysis of transgenic *Arabidopsis thaliana* lines for these genes revealed a clear phenotype of early senescence in line HaNAC001- [4]. This line also showed a stronger *HaNAC001* expression by qPCR assay.

OG-1 h was an OG functional related to the senescence process in *Arabidopsis thaliana*. For example, genes such as ORE1 and ANAC100 belong to this group (Additional files 3 and 8). Interestingly, ORE1 is a key regulator that triggers senescence under the control of the ethylene signaling pathway (He et al., 2005; Jin et al., 2009). In sunflower, OG-1 h had six paralogs (HaNAC001, HaNAC046, HaNAC077, HaNAC126, HaNAC129 and HaNAC142) with a typical 3 exon structure with the complete NAC domain (subdomains A-E) positioned in the N-terminal. These genes lacked unique motifs, extra domains or transmembrane domains.

According to the RNAseq analysis, three of the six genes (HaNAC001, HaNAC077, HaNAC126) have a differential expression profile. Two of them were upregulated, whereas HaNAC001 was downregulated in line R453 vs B481–6 in anthesis. This finding was in contrast to the expected results and to a previous study [19]. Since R453 has a premature senescence phenotype, an overexpression of this last gene, as a trigger of senescence, was likely. However, the previous research with opposite results studied other sunflower genotypes and at different developmental stages. Even more, ORE1 is an early regulator of the senescence in Arabidopsis. In the present study, the expression of HaNAC001 remained similar at anthesis and post anthesis in the R453, it can therefore be expected that the expression of this gene was previous to anthesis. Whereas the expression of HaNAC001 in the B481–6 line was not significantly upregulated between post anthesis and anthesis. This result suggested that HaNAC001 had delayed expression in the B481–6 line when it is compared with R453, in accordance with the senescence-delayed phenotype. Besides, the early senescence phenotype observed here in one of the Arabidopsis transgenic lines (Fig. 8), pointed that HaNAC001 could trigger senescence in sunflower before the anthesis, as was evidenced by the result reported for ORE1 [41]. Hence, HaNAC077 and HaNAC126 may have similar functionality as triggers of senescence in a more delayed sense.

HaNAC003 and HaNAC005 belong to OG-NAC-3e, similarly to the *A. thaliana* genes ANAC019, ANAC072, and ANAC055. These genes belong to the same clade of NAC genes and have overlapping expression patterns [42]. However, ANAC072 shows different regulation networks compared to ANAC055 and ANAC019, depending on the stimuli [43, 44].

HaNAC007 belongs to OG-NAC-3c. In fact, NAP is an Arabidopsis NAC TF of OG-NAC-3c; this gene is well known for its regulation of senescence dependent on ABA and ethylene signalling [45]. In the present study, HaNAC007 had a downregulation profile during post-anthesis in the B481–6 line, whereas it had an upexpression pattern in R453 (premature senescence phenotype). These results support HaNAC007 as a positive regulator of senescence. On the other hand, regulation sequences of this OG are characterized by the presence of the Myb/SANT and NF-YB TFBS, which are important transcription factors in the regulation of senescence in sunflower [19] and Arabidopsis [46], respectively. In this study, HaNAC007 showed no significant change in the Arabidopsis transgenic lines. But we believe that this result can be a consequence to post-traductional regulation of the transgene. Taken together, further studies with large number of transgenics lines and proteomic experiments on the HaNAC proteins are needed to clarify the implication of this proteins in the regulation networks of sunflower senescence.

## Conclusions

The genome analysis of the sunflower NAC genes, along with their classification in OGs, is a useful tool to facilitate comparisons with NAC genes in other species with the purpose of inferring gene functions. Besides, regularity sequences and motifs analysis are useful for establishing gene networks with other TFs. In this research, we assessed the functionality of four HaNAC genes: *HaNAC001*, *HaNAC003*, *HaNAC005* and *HaNAC007*; and at least one of them seems to be a real regulator of senescence. Other interesting genes probably associated with senescence emerge from this study: HaNAC044, HaNAC047, HaNAC082, HaNAC098 and HaNAC146.

## Methods

### Orthologous groups of NAC genes in *H. annuus* genome

For all the analysis *H. annuus* genome HanXRQr2, INRAE Sunflower Bioinformatics Resources was used [30, 47]. A survey of the *H. annuus* genome for presence of NAC genes was performed through a tBLASTn analysis using *Vitis vinifera* NAC sequences. NAC amino acid sequences were then assigned to the respective orthogroup (OG) based on BLASTp hits on the protein database containing the whole set of NAC genes of *Musa acuminata*, *Oryza sativa*, *V. vinifera* and *Arabidopsis thaliana* [28]*.* Forty OGs were defined by Cenci et al. [28]: 1a-1 h, 2a-2f, 3a-3e, 4a-4 g, 5a-5c, 6a-6c, 7a-7f, 8a, 8b. If at least, the three best BLASTp hits belong to the same previously established OG-NAC, then the *H. annuus* NAC gene was assigned to this OG.

### Phylogenetic analysis

NAC amino acid sequences of *H. annuus* and the four species representative of angiosperms were aligned with the MUSCLE program [48] via the EMBL-EBI bioinformatics interface [49] using the default parameters. Conserved blocks were extracted from the alignments with Gblocks [50]. The analysis was performed by allowing (i) smaller final blocks, (ii) gap positions within the final blocks, and (iii) less strict flanking positions. Phylogenetic trees were built with PhyML [51] available at [52] using an LG substitution model and an Approximate Likelihood-Ratio Test (aLRT) as statistical tests for branch support. The obtained phylogenetic trees were visualized with MEGA6 [53].

### Exon/intron structure analysis

HaNAC sequences (CDS and genomic) were extracted from the INRAE Sunflower Bioinformatics Resources to analyze them with the GSDS (Gene Structure Display Server) and visualize exon/intron structure of the HaNAC genes [54].

### Chromosomal location and gene duplication analysis

The gene sequences and their location in the genome were obtained from the sunflower genome database. A genomic map including the location of NAC genes was drawn using the KaryoploteR R package. Eight HaNAC were named as reported previously (HaNAC001, HaNAC002, HaNAC003, HaNAC004, HaNAC005, HaNAC006, HaNAC007 and HaNAC008) [55]. All genes were numbered successively according to their position in the genome, except for five genes (HaNAC147, HaNAC148, HaNAC149, HaNAC150, and HaNAC151), which were manually annotated. The NAC genes of the same OG that were separated by no more than 100,000 bp were considered as tandem duplications.

### Domain and motif structure in the sunflower NAC family

Amino acid lengths, molecular weights, isoelectric points and other physicochemical features of the NAC proteins were obtained using the EMBOSS pepstats and the ExPASy protparam programs.

The 151 coding sequences were analyzed in search of transmembrane domains with the TMHMM tool and other Pfam entry using the default parameters in both cases. The evaluation of protein motifs was carried out using MEME with the following parameters: motif width set to 6–200 and number set to 10 [34]. The distribution of motifs along the 151 proteins was grouped in four different structures (A, B, C, D). Finally, sequence alignment comparisons of the subdomains (A-E) in search of NAC subdomains [26, 56] in the coding sequences [57] were performed by using ClustalW [58].

### Regulatory elements of NAC genes in sunflower

The differences in the promoter region of the HaNAC were evaluated using the database PlantPAN 3.0, which contained 17,230 TFs and 4703 matrices of TF binding sites among 78 plant species at the time of this analysis. The analysis consisted of looking for already described transcription factor binding sites. For this purpose, 2000-bp sequences upstream of the ATG were extracted from the Heliagene database and analyzed with the tool Multiple Promoter Analysis from the PlantPLAN 3.0 database. We obtained a frequency value of each TFBS for the 151 HaNAC promoters. Then the TFBS mean was calculated for OGs. The mean values were plotted in a heatmap using ggplot2 R package [35].

### Gene expression experiment of two contrasting lines

The gene expression analysis for all the sunflower NAC genes was based on an RNAseq to assess senescence-associated traits in contrasting lines [18]. The experiment was conducted under field conditions at the Biotechnology Institute INTA Castelar in the 2014/15 growing season. The inbred lines used in this study, which were previously selected from the INTA Sunflower Breeding Program, INTA Manfredi Sunflower Germplasm Collection, display contrasting senescence traits: R453 (early senescence) and B481–6 (delayed senescence) [59]. Each analysis consisted of three biological replicates (plots), each one coming from three randomly selected plants. Soil fertility assured maximum yields under non-limiting water conditions, which were maintained by irrigation. Transcriptomic profiles were performed using the 15th leaf (numbered from the bottom to the top of the plant) at two developmental stages, anthesis and post-anthesis time (12 days after anthesis). High quality total RNA was isolated from leaves. The purity and integrity of total RNA were determined by FragmentAnalyzer (Advanced Analytical Technologies, USA).

RNA libraries were generated using the kit TruSeqStranded mRNA Technology (Illumina) and evaluated according to standard quality controls of the Argentine Consortium of Genomic Technologies (CATG, by its acronym in Spanish). Each library was paired-end sequenced (2 × 100 bp) with a depth of 20 million reads [60] using a HiSeq-1500 (Illumina) (NCBI - SRA accession: PRJNA525834).

### RNA-seq data analysis

The data obtained in a previous study [18] were analyzed using the new version of sunflower genome. The quality control of reads was evaluated using FastQC and trimmed or filtered using trim_galore [61]. The obtained reads were aligned to the reference genome (30) using Bowtie2 software with the following parameters: “global alignment” and “to keep all multi-mapping reads”. All the obtained read mapping files (BAM files) were used to perform re-estimation of all counts to all transcripts using eXpress software [62], which uses an EM method for the estimation of multi-mapping reads. A statistical and differential gene expression analysis was performed using the DESeq2 package [63] in R.

### Transgenic lines

The complete sequence of each of the selected genes (HaNAC001, HaNAC003, HaNAC005 and HaNAC007) was isolated and cloned in a GATEWAY binary vector under the promoter of the small Rubisco subunit (ribulose 1,5 bisphosphate carboxylase oxygenase). Each construct was incorporated into *Agrobacterium tumefaciens* (strain GV3101) and used to transform Arabidopsis plants (Col-0 ecotype) and two independent lines were selected for each gene construct (HaNAC01–1, HaNAC01–4, HaNAC03–3, HaNAC03–5, HaNAC05–1, HaNAC05–5, HaNAC07–2, HaNAC07–5).

The expression levels of these transgenes were characterized by quantitative PCR (qPCR) and the analyses revealed that each HaNAC construct displayed different expression levels of the transgene for each independent line. Particularly, HaNAC01–4, HaNAC03–5, HaNAC05–5 and Ha-NAC07–5 showed high expression profiles for the transgenes (Additional file 10). The expression level of the transgenic lines was evaluated by qPCR using Elongation Factor 1a (EF-1a) as a reference gene, as described previously [64].

### Phenotyping assays

The phenotype of transgenic lines was quantified in the high throughput phenotyping platform PHENOPSIS [65]. Seeds were kept in the dark at 4 °C for at least 1 week before sowing. Four to six seeds were sown on the surface of the soil of 225-ml pots filled with a mixture of loamy soil and organic compost (Neuhaus N2). The soil surface was moistened with one-tenth-strength Hoagland solution and the pots were kept in the dark for 48 h under controlled environmental conditions (20.5 °C and 70% air relative humidity). Then, the pots were placed in the PHENOPSIS growth chamber at 20.8 °C with a 16-h-light photoperiod, 190 μmol m^− 2^ s^− 1^ photosynthetic photon flux density, 70% air relative humidity, 0.7 kPa water vapor pressure deficit of water vapor. The pots were sprayed with deionized water three times a day until germination. The water content of the soil in each pot was adjusted to 0.35 g water g^− 1^ dry soil after germination. After the emergence of the first two true leaves (stage 1.02) [66], the soil water content was automatically maintained at 0.35, of water g-1 dry soil, by daily adjustment with one tenth-strength Hoagland’s nutrient solution. After the emergence of the fourth leaf in one of the plants, the others were removed from the pot; in this way, each pot contained only one plant, thus giving twelve biological replicates per accession. Visible light images of each pot were taken automatically once a day throughout the experiment.

### Morpho-physiological traits

Several growth and morpho-physiological traits were measured at flowering stage (i.e. first visible open flower) for each Arabidopsis transgenic lines and Col-0: number of senescent leaves, leaf number, rosette fresh weight (mg), rosette dry weight (mg), total leaf area (mm^2^), Hue pixel ratio (40–61).

Rosette fresh weight was obtained immediately after harvest. Leaf laminas were detached from the rosette, counted and scanned to determine total leaf area using ImageJ software [67]. Then, lamina and rachis dry weights (rosette dry weight) were separately obtained after drying for 72 h at 80 °C. Hue pixel ratio (40–61) was determined by using the Matlab Color App Thresholder tool of the Image Processing Toolbox R2019a (MATLAB, Mathworks, Natick, MA, USA). Hue pixel ratio (40–61) is the ratio of pixel number with a hue value between 40 and 61, and total pixel number, measured for all the leaves of each plant. This ratio is an indicator of the proportion of senescing areas. Two additional ratios were calculated: the dry matter content (rosette dry weight / rosette fresh weight) and the leaf mass area (total leaf dry weight / total leaf area).

The images taken by PHENOPSIS, also, were processed using ImageJ software [67] to extract projected rosette area (mm^2^) through time. Growth duration (d) and maximum expansion rate (mm^2^ d^− 1^) were extracted from the model parameters after fitting a sigmoid curve to individual projected areas with the gcFitModel function from the R/grofit package [68].

Leaf number, rosette dry weight (mg), total leaf area (mm^2^), Hue pixel ratio (40–61), dry matter content, leaf mass area, growth duration and maximum expansion rate were used as active variables in a principal component analysis (PCA) performed in R.

## Supplementary Information

**Additional file 1.** Sunflower NAC TF. Identified Sunflower NAC TF in Heliagene database and their orthologs groups based on phylogenetic studies of NAC proteins sequences. The table contains information about the transmembrane domains and other non NAC domains for each gene.

**Additional file 2. **Phylogenetic trees. The amino acid sequences of the NAC proteins of *Arabidopsis thaliana, Vitis vinifera, Musa acuminata*, *Oryza sativa* and *Helianthus annuus* were used to reconstruct a phylogenetic tree. Phylogenetic trees were built with PhyML available at using an LG substitution model and an Approximate Likelihood-Ratio Test (aLRT) as statistical tests for branch support. The obtained phylogenetic trees were visualized with MEGA6.

**Additional file 3. **Phylogenetic trees. The amino acid sequences of the NAC proteins of *Arabidopsis thaliana, Vitis vinifera, Musa acuminata*, *Oryza sativa* and *Helianthus annuus* were used to reconstruct a phylogenetic tree. Phylogenetic trees were built with PhyML available at using an LG substitution model and an Approximate Likelihood-Ratio Test (aLRT) as statistical tests for branch support. The obtained phylogenetic trees were visualized with MEGA6.

**Additional file 4. **Phylogenetic trees. The amino acid sequences of the NAC proteins of *Arabidopsis thaliana, Vitis vinifera, Musa acuminata*, *Oryza sativa* and *Helianthus annuus* were used to reconstruct a phylogenetic tree. Phylogenetic trees were built with PhyML available at using an LG substitution model and an Approximate Likelihood-Ratio Test (aLRT) as statistical tests for branch support. The obtained phylogenetic trees were visualized with MEGA6.

**Additional file 5. **Phylogenetic trees. The amino acid sequences of the NAC proteins of *Arabidopsis thaliana, Vitis vinifera, Musa acuminata*, *Oryza sativa* and *Helianthus annuus* were used to reconstruct a phylogenetic tree. Phylogenetic trees were built with PhyML available at using an LG substitution model and an Approximate Likelihood-Ratio Test (aLRT) as statistical tests for branch support. The obtained phylogenetic trees were visualized with MEGA6.

**Additional file 6. **Phylogenetic trees. The amino acid sequences of the NAC proteins of *Arabidopsis thaliana, Vitis vinifera, Musa acuminata*, *Oryza sativa* and *Helianthus annuus* were used to reconstruct a phylogenetic tree. Phylogenetic trees were built with PhyML available at using an LG substitution model and an Approximate Likelihood-Ratio Test (aLRT) as statistical tests for branch support. The obtained phylogenetic trees were visualized with MEGA6.

**Additional file 7.** Gene structure analysis. Visualization of intron exon NAC genes analysis by GSDS (Gene Structure Display Server).

**Additional file 8.** ARNseq data. Expression data of Sunflower NAC TF of two contrasting lines at two different times (anthesis and post-anthesis).

**Additional file 9. **Bibliography of *Arabidopsis thaliana* NAC TF associated with leaf senescence.

**Additional file 10.** Transgene expression analysis. The expression levels of HaNAC01–1, HaNAC01–4, HaNAC03–3, HaNAC04–5, HaNAC05–1, HaNAC05–5, HaNAC07–2, HaNAC07–5 transgenes were measured by quantitative PCR (qPCR) using Elongation Factor 1a (EF-1a) as a reference gene, as described previously.

## Data Availability

All data generated or analysed during this study are included in this published article and its additional files. RNAseq data are available at NCBI opensource database, SRA accession: PRJNA525834. https://www.ncbi.nlm.nih.gov/bioproject/PRJNA525834

## Abbreviations

A: Anthesis
aLRT: Approximate likelihood-ratio test
FT: Transcription factor
NAC: NAM, ATAF and CUC domains
OG: Orthologous Group
PA: Post-anthesis
TFBS: Transcription factor binding site
TRR: Transcription regulatory regions
WGD: Whole genome duplication
