# Correction to: Genome-wide and comparative phylogenetic analysis of senescence-associated NAC transcription factors in sunfower (Helianthus *annuus*)

**DOI:** 10.1186/s12864-021-08270-1

**Published:** 2022-01-04

**Authors:** Sofa A. Bengoa Luoni, Alberto Cenci, Sebastian Moschen, Salvador Nicosia, Laura M. Radonic, Julia V. Sabio y García, Nicolas B. Langlade, Denis Vile, Cecilia Vazquez Rovere, Paula Fernandez

**Affiliations:** 1 Instituto de Agrobiotecnología y Biología Molecular, INTA-Castelar, Buenos Aires, Argentina; 2 Bioversity International, Montpellier, France; 3 Estación Experimental Agropecuaria Famaillá, INTA-Famaillá, Tucumán, Argentina; 4 Université de Toulouse, INRAE, CNRS, Castanet-Tolosan, France; 5 LEPSE, Université de Montpellier, INRAE, Montpellier, France

**Correction to: BMC Genomics 22, 893 (2021)**

**https://doi.org/10.1186/s12864-021-08199-5**

Following publication of the original article [1], an error was reported in the authorship panel. The name of Julia V. Sabio y García was incorrectly written as ‘Julia Sabio Garcia’.

The original article has been corrected.
