# Supplementary material for: Genome-wide and comparative phylogenetic analysis of senescence-associated NAC transcription factors in sunflower (Helianthus annuus)
Source: BMC Genomics. 2021 Dec 14;22:893. doi: 10.1186/s12864-021-08199-5 (PMC8670195; doi:10.1186/s12864-021-08199-5)

Genome-Wide and Comparative Phylogenetic Analysis of Senescence-Associated NAC Transcription Factors in Sunflower (H*elianthus annuus*)

Bengoa Luoni Sofia A., Cenci Alberto, Moschen Sebastian, Nicosia Salvador, Radonic, Laura M., Sabio y Garcia Julia, Langlade Nicolas B., Vile Denis, Vazquez Rovere Cecilia and Fernandez Paula.

Additional file 3: Phylogenetic tree

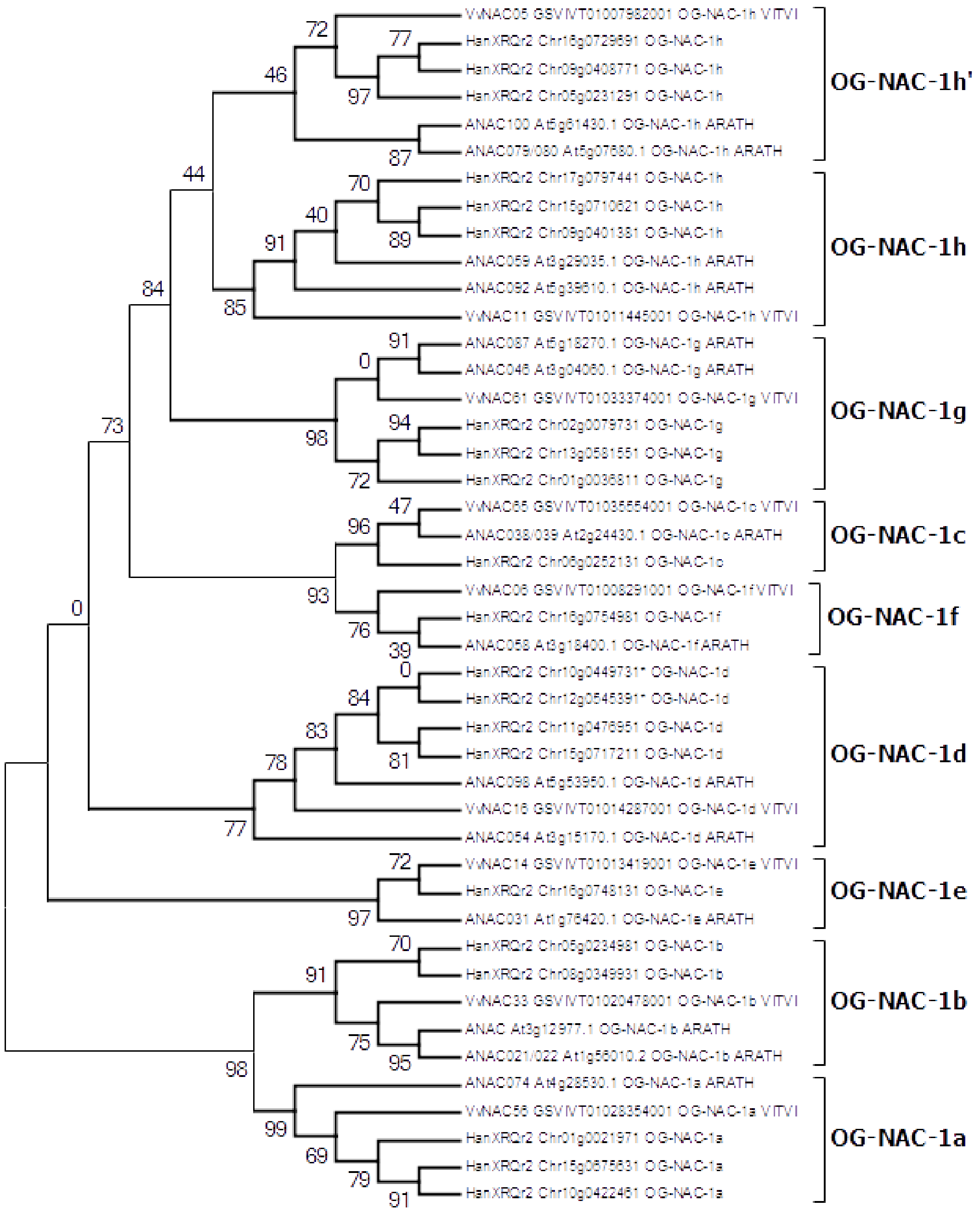

Supplement: Supplementary file 3 — Additional file 3. Phylogenetic trees. The amino acid sequences of the NAC proteins of Arabidopsis thaliana, Vitis vinifera, Musa acuminata, Oryza sativa and Helianthus annuus were used to reconstruct a phylogenetic tree. Phylogenetic trees were built with PhyML available at using an LG substitution model and an Approximate Likelihood-Ratio Test (aLRT) as statistical tests for branch support. The obtained phylogenetic trees were visualized with MEGA6. [file 12864_2021_8199_MOESM3_ESM.pdf]
