# Supplementary material for: Genome-wide and comparative phylogenetic analysis of senescence-associated NAC transcription factors in sunflower (Helianthus annuus)
Source: BMC Genomics. 2021 Dec 14;22:893. doi: 10.1186/s12864-021-08199-5 (PMC8670195; doi:10.1186/s12864-021-08199-5)

Genome-Wide and Comparative Phylogenetic Analysis of Senescence-Associated NAC Transcription Factors in Sunflower (*Helianthus annuus*)

Bengoa Luoni Sofia A., Cenci Alberto, Moschen Sebastian, Nicosia Salvador, Radonic, Laura M., Sabio y Garcia Julia, Langlade Nicolas B., Vile Denis, Vazquez Rovere Cecilia and Fernandez Paula.

Additional file 7a: Gene structure

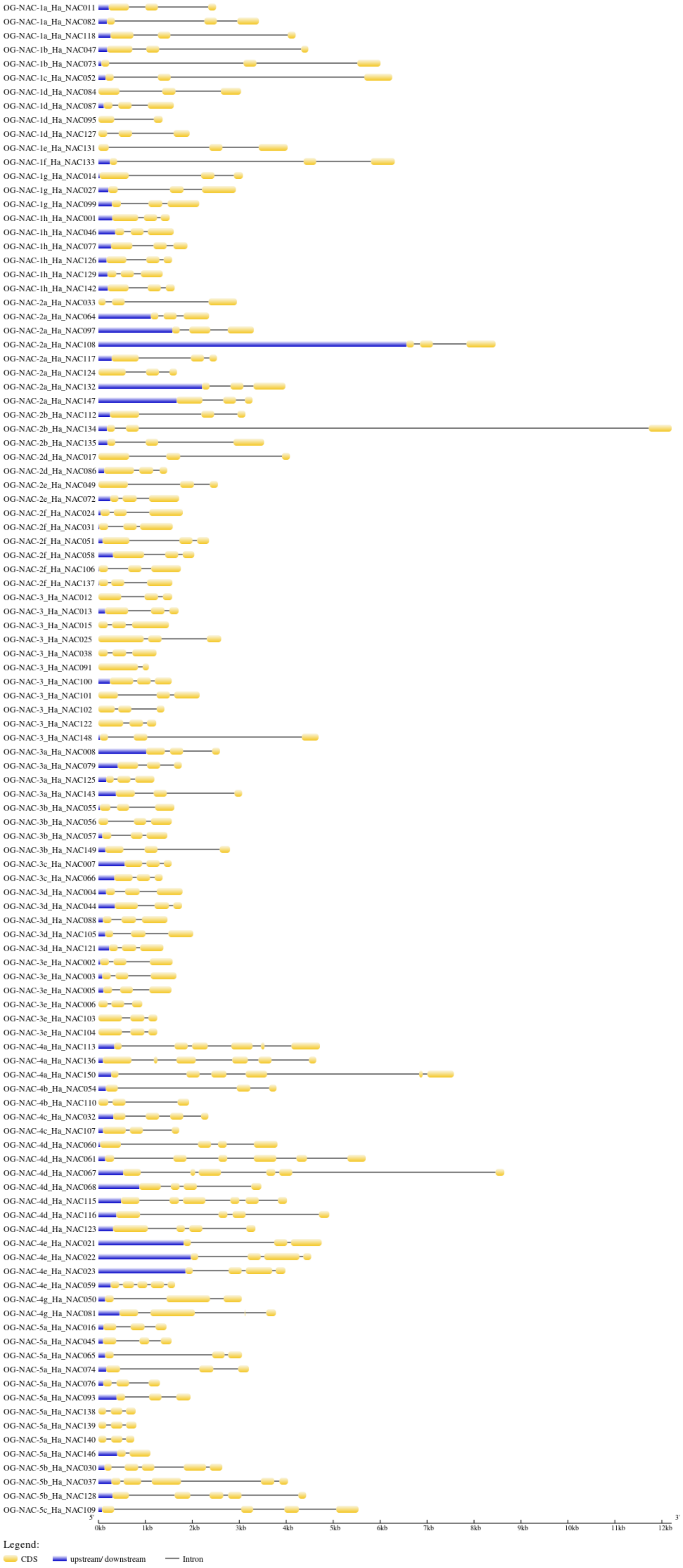

Additional file 7b: Gene structure

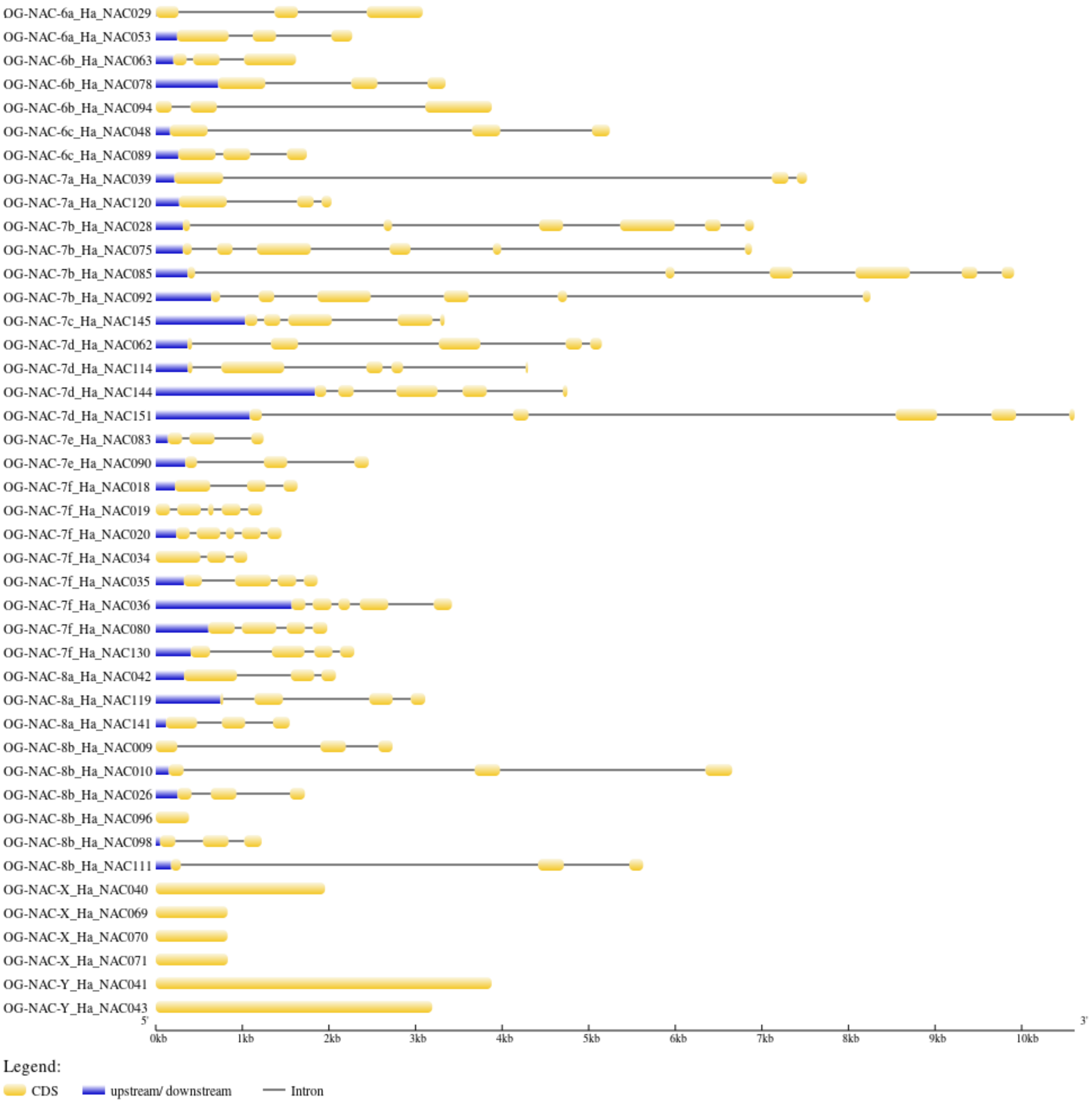

Supplement: Supplementary file 7 — Additional file 7. Gene structure analysis. Visualization of intron exon NAC genes analysis by GSDS (Gene Structure Display Server). [file 12864_2021_8199_MOESM7_ESM.pdf]
