# Supplementary material for: Genome-wide and comparative phylogenetic analysis of senescence-associated NAC transcription factors in sunflower (Helianthus annuus)
Source: BMC Genomics. 2021 Dec 14;22:893. doi: 10.1186/s12864-021-08199-5 (PMC8670195; doi:10.1186/s12864-021-08199-5)

Genome-Wide and Comparative Phylogenetic Analysis of Senescence-Associated NAC Transcription Factors in Sunflower (H*elianthus annuus*)

Bengoa Luoni Sofia A., Cenci Alberto, Moschen Sebastian, Nicosia Salvador, Radonic, Laura M., Sabio y Garcia Julia, Langlade Nicolas B., Vile Denis, Vazquez Rovere Cecilia and Fernandez Paula.

Additional file 10: Transgene expression analysis.

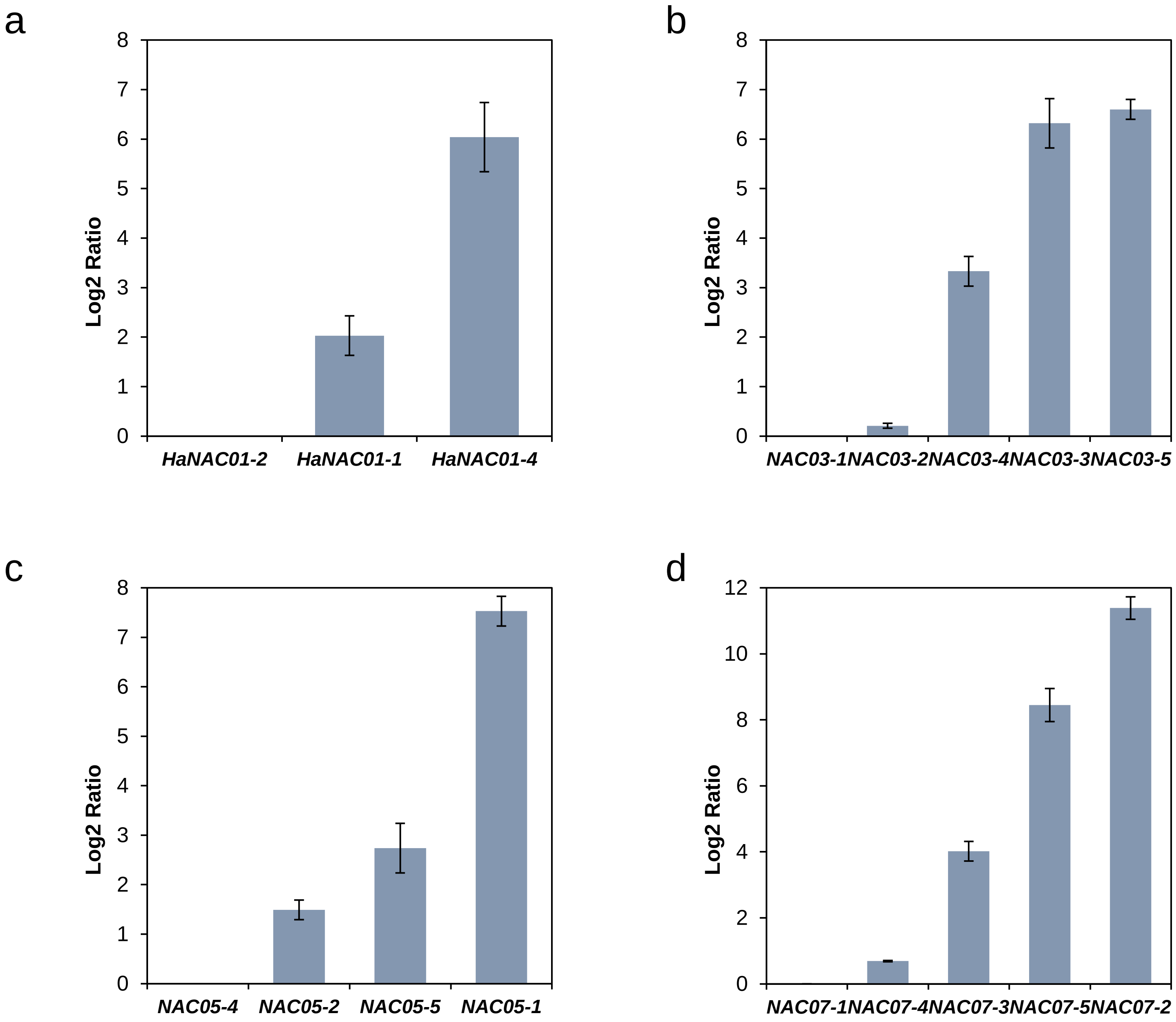

Supplement: Supplementary file 10 — Additional file 10. Transgene expression analysis. The expression levels of HaNAC01–1, HaNAC01–4, HaNAC03–3, HaNAC04–5, HaNAC05–1, HaNAC05–5, HaNAC07–2, HaNAC07–5 transgenes were measured by quantitative PCR (qPCR) using Elongation Factor 1a (EF-1a) as a reference gene, as described previously. [file 12864_2021_8199_MOESM10_ESM.pdf]
